# Differential Behavior of Uncleaved gp160 Human Immunodeficiency Virus Type 1 (HIV-1) Envelope (Env) Glycoproteins

**DOI:** 10.64898/2026.10.14.759227

**Published:** 2026-10-16

**Authors:** Stephanie M. Sauve, Mahmoud Moradi

## Abstract

The envelope glycoprotein (Env) of human immunodeficiency virus type 1 (HIV-1) plays important roles in viral entry by coordinating host-cell receptor recognition and membrane fusion. Env is synthesized as a homotrimeric precursor, gp160, which is cleaved into gp120 and gp41 subunits. Although the cleaved form of Env is required for fusion, uncleaved Env remains plentiful on the surface of HIV-1-infected cells. Previous studies have suggested that uncleaved Env is conformationally more flexible than the cleaved form making its dynamics important for understanding the broader structural landscape of HIV-1 Env. In this study, we employed microsecond-long all-atom molecular dynamics (MD) simulations to characterize the dynamics of two structures of uncleaved HIV-1 Env that have been previously assigned to the same conformational state. Our results reveal differences in their conformational behavior, including differences in collective motions, FRET distributions, and trimer symmetry. These findings provide molecular-level insight into the structural plasticity of uncleaved HIV-1 Env and highlight that different structures within the same conformational state can exhibit distinct dynamic behavior.

